# Prevention and Periodontal Treatment in Down Syndrome Patients: A Systematic Review

**DOI:** 10.1371/journal.pone.0158339

**Published:** 2016-06-29

**Authors:** Rafael Ferreira, Raphaella Coelho Michel, Sebastião Luiz Aguiar Greghi, Maria Lúcia Rubo de Resende, Adriana Campos Passanezi Sant’Ana, Carla Andreotti Damante, Mariana Schutzer Ragghianti Zangrando

**Affiliations:** Division of Periodontics, Department of Prosthodontics, Bauru School of Dentistry, University of São Paulo, Bauru, SP, Brazil; Boston University Goldman School of Dental Medicine, UNITED STATES

## Abstract

The aim of this systematic literature review was to evaluate which type of periodontal preventive and therapeutic approaches presents superior outcomes in patients with Down syndrome (DS). Studies reporting different methods of periodontal care from DS patients were considered eligible. Included clinical studies should indicate at least two periodontal parameters in different periods of assessment. Screening of the articles, data extraction and quality assessment were conducted independently and in duplicate. Electronic search according to the PICO search, with both Key-words and MESH terms were conducted in MEDLINE, EMBASE and CENTRAL databases until March 2016. Manual search was conducted in four journals, namely Journal of Periodontology, Journal of Clinical Periodontology, Journal of Periodontal Research and Special Care in Dentistry and their electronic databases were searched. Electronic and manual search resulted in 763 papers, and of them 744 were excluded after title/abstract assessment. The full text of 19 potentially eligible publications was screened and 9 studies met inclusion criteria. The results demonstrated the importance to introduce youngest DS patients in preventive programs, as well as participation of parents, caregivers or institutional attendants in supervising/performing oral hygiene. In studies with higher frequency of attendance, all age groups presented superior preventive and therapeutic results, irrespective of the therapeutic approach used (surgical/nonsurgical/periodontal care program). The important factors for reducing periodontal parameters were the frequency of the appointments and association with chlorhexidine/plaque disclosing agents as adjuvant treatment. This systematic review demonstrated that early introduction in periodontal care, participation of parents/caregivers/institutional attendants, frequency of attendance and association with chemical adjuvants (independently of the periodontal treatment adopted) seems to improve periodontal outcomes in preventive and periodontal treatment of DS patients. Registration number (Prospero): CRD42016038433.

## Introduction

Down syndrome (DS) is an autosomal chromosomal anomaly associated with trisomy of chromosome 21 [1]. It is characterized by the whole chromosomal aneuploidy in about 95% of cases. The remaining 5% is in the form of translocations and mosaics [2]. DS is the most common genetic birth defects, affecting approximately one in 700 live births [3,4,5]. According to National Down Syndrome Society (NDSS) [6], more than 400.000 individuals with DS live in the United States. Moreover, life expectancy for DS patients increased dramatically in recent decades, from 25 years in 1983 to 60 years today [6].

DS individuals present anatomical abnormalities, mental and orofacial problems that present a large impact in quality of life [7]. Furthermore, DS patients are more susceptible to infections including an increased prevalence of periodontal diseases, almost 100% under the age of 30 years [8,9]. Periodontal disease in these patients is severe, generalized, with rapid progression and classified as a manifestation of systemic diseases associated with genetic disorders by American Academy of Periodontolgy [10]. Poor oral hygiene *per se* may not explain severe and generalized periodontal destruction observed in DS patients. This condition is also associated with impairment of immunological system [11–15]. DS patients present mild to moderately reduced T and B cell counts, absence of normal lymphocyte expansion in infancy, suboptimal antibody responses to immunizations, decreased immunoglobulin A in saliva and neutrophil chemotaxis [14]. Current data showed a high level of TNF-α and IFN-γ in children with DS, these inflammatory cytokines present biological effects in the body and important regulatory roles in immune responses [13]. Another study demonstrated altered expression of immune-related genes in children with DS, highlighting molecular mechanisms involved in DS pathology [16]. Moreover, some local disorders are related to the development of early periodontal disease, such as poor occlusal correlation, high frenum insertion, early mucogingival problems and advanced tongue position. In addition to periodontal treatment, DS patients must receive attention and management of dental caries [17,18], malocclusion [19] and obstructive sleep apnea [20].

Preventive approaches and treatment modalities of gingivitis and periodontitis include removal of dental biofilm, surgical and nonsurgical therapy. Preventive actions involve supervised brushing or stimulation of oral hygiene habits. Periodontal treatment basically include scaling and root planing (surgical or non-surgical), associated or not with local and/or systemic antibiotics [21–23]. Furthermore, participation of parents, caregivers and possibly institutional attendants are fundamental for the maintenance of outcomes achieved.

Therefore, preventive methods and conventional periodontal treatments usually present disappointing outcomes in this group of patients, requiring specific approaches. These factors justify the necessity of further research efforts including more effective preventive and treatment therapies and possible association with adjunctive chemical substances [8]. A systematic review [24] reported that patients with intellectual disabilities presented poorer oral hygiene and more severe periodontal destruction than control patients. Considering the higher prevalence of periodontal disease and the evident necessity of prevention and periodontal treatment in DS population, lack of systematic review on this topic justifies the present study.

The aim of this systematic review was to evaluate which type of approach—preventive programs and different periodontal therapies—presents improved periodontal outcomes for DS patients. The following focused question was addressed: “Which type of periodontal preventive and therapeutic approaches presents superior periodontal outcomes in DS patients?”

## Materials and Methods

This review was conducted in accordance with PRISMA [25], the Cochrane Collaboration [26] and Check Review [27] guidelines (PROSPERO Registration number: CRD42016038433).

### Selection Criteria

- Inclusion criteria: Longitudinal and observational studies, controlled clinical trials, randomized clinical trials and case series; studies reporting different methods of periodontal preventive care and periodontal treatment in DS patients; studies should indicate at least two periodontal parameters in different periods of assessment.
- Exclusion criteria: Transversal studies, pilot studies, literature review, studies without description of detailed periodontal care or treatment and periodontal parameters, absence of statistical analysis.

### Outcome Measure

Primary–evaluation of periodontal parameters related to presence or absence of plaque, calculus and gingival inflammation, i.e, bleeding on probing (BOP), plaque index (PI), gingival index (GI);

Secondary–probing depth (PD), clinical attachment level (CAL), radiographic bone loss (BL).

### Search Strategy

**Fig 1:**
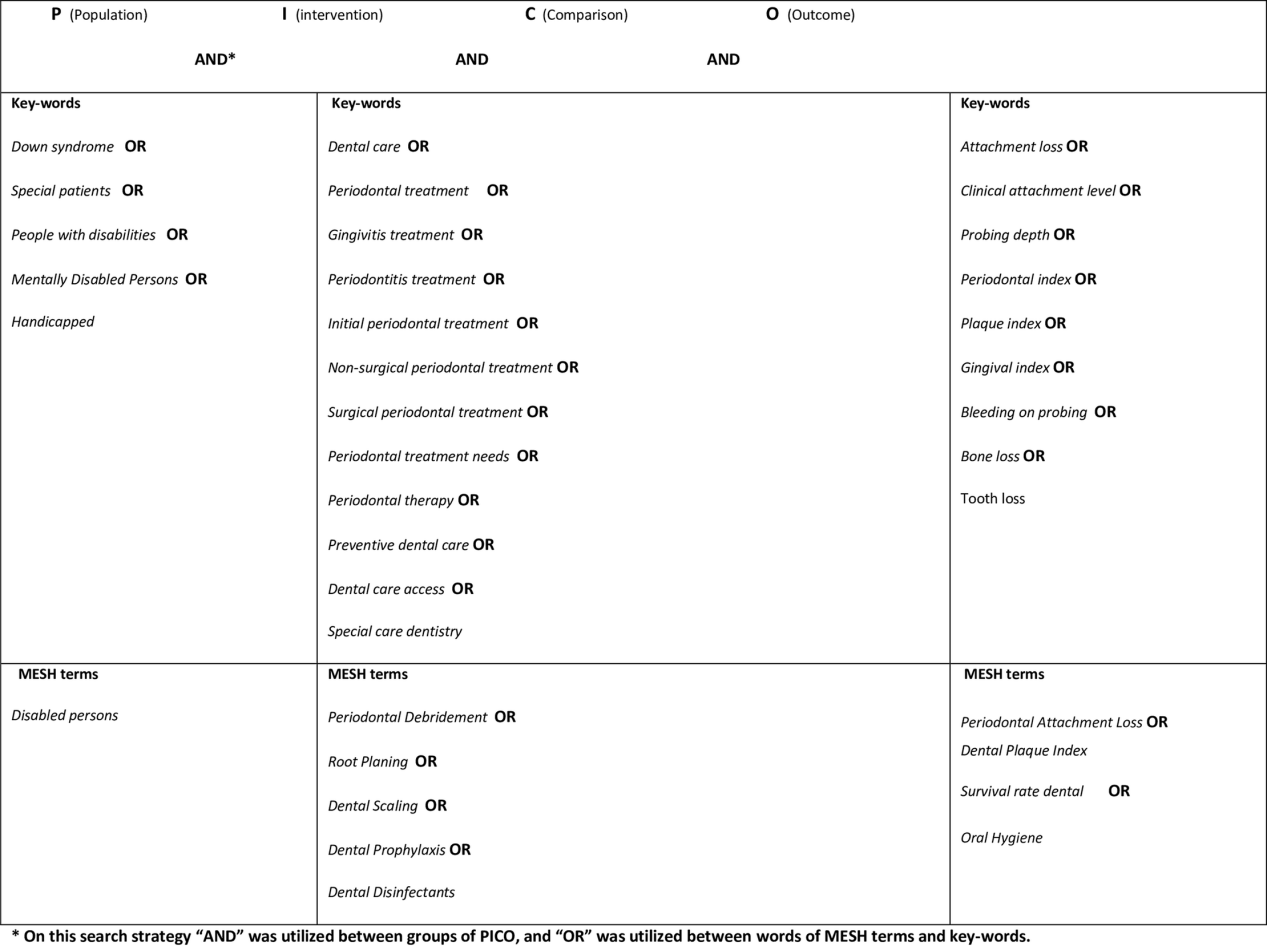
Electronic search according to the PICO scheme, with both Key-words and MESH terms.

Comprehensive search strategies were established to identify studies for this systematic review. The MEDLINE, EMBASE and CENTRAL databases were searched for papers published until March 2016. Electronic search without language restrictions were conducted according to the PICO scheme (Fig 1), with both Key-words and MESH terms ((((Special patients OR Disabilities OR Mentally Disabled Persons OR Handicapped OR Down syndrome) AND (Periodontal treatment OR Gingivitis treatment OR Non-surgical periodontal treatment OR Surgical periodontal treatment OR Preventive dental care OR Special care dentistry OR Dental care OR Periodontal Debridement OR Dental care for handicapped OR Dental care for children OR Dental Care for Disabled) AND (Clinical attachment level OR Probing depth OR Gingival index OR Periodontal probe OR Periodontal index OR Periodontal Attachment Loss OR Dental Plaque Index) AND (Successful rate OR Education dental hygiene OR Education dental health OR Survival rate dental OR Health Education Dental OR Comprehensive Dental Care OR Oral Hygiene OR Professional Practice)))). Reference lists of any potential articles were examined and unpublished studies were identified by searching the OpenGREY database. Manual search also included four journals (Journal of Periodontology, Journal of Clinical Periodontology, Journal of Periodontal Research and Special Care in Dentistry) identified as important to review, and their electronic databases were searched.

### Assessment of validity and data extraction

Two independent reviewers (MSRZ and RF) evaluated titles, abstracts and full texts considering the search strategy of the identified papers. Disagreement between the reviewers was resolved through discussion until consensus was reached. When agreement could not be reached, a third reviewer (CAD) was consulted. The following data were extracted and recorded in duplicate: (1) citation, publication status and year of publication; (2) study design; (3) characteristics of the participants and procedures (intervention groups, dental care program, periodontal treatment); (4) main results and (5) conclusions.

### Quality assessment and risk of bias in included studies

Two methodological quality assessment tools were used on the basis of the type of study. For randomized and controlled clinical trials, methodological quality of the trials was evaluated using a Cochrane Collaboration’s tool for assessing risk of bias [26], as adapted by Chambrone et al. 2010a [27]. Concisely, the methods used for randomization and allocation were classified in adequate, inadequate, unclear, or not applicable. Blinding of examiners and completeness of the follow-up period were registered with yes/no responses. Considering these answers, risk of bias was categorized in: (1) low risk of bias- all criteria were met (i.e., randomization and allocation concealment were correctly described and positive answers to all questions about completeness of follow-up questions and masking of examiners), (2) unclear risk of bias- one or more criteria were partly met (i.e., unclear criteria were set), or (3) high risk of bias- one or more criteria were not met.

The methodological quality of observational studies was performed with the assistance of an adapted version [28–32] of the Newcastle-Ottawa scale (NOS) [33]. The following topics were evaluated: selection of study groups (sample size calculation, representativeness of the DS patients, and selection of the health patients), ascertainment or assessment of periodontal conditions, clarity in the description of dental care program or periodontal treatment, training or calibration of assessors of outcomes, data collection methods, and use of clear inclusion/exclusion criteria; comparability (comparability of patients based on study design/analysis and management of confounders); outcome (evaluation of results, ascertainment or criteria applied to confirm periodontal parameters, and adequacy of patient follow-up); and statistical analysis (appropriateness/validity of statistical analysis and unit of analysis reported). Additionally, stars or points were given for each methodological quality criterion and each included study could receive a maximum of 14 points. Studies with 11–14 points (approximately 80% or more of the domains satisfactorily fulfilled) were arbitrarily considered to be of high quality, studies with 8–10 stars were of medium quality, and studies with <8 stars were of low methodological quality.

### Data Synthesis

The data were organized into evidence tables and a summary of study design, sample, procedures, outcomes and quality of evidence were described. Preventive periodontal care included studies with different preventive approaches without professional scaling and root planing (Table 1) and periodontal treatments included surgical and nonsurgical procedures associated or not with chemical agents (Table 2).

**Table 1:** Characteristics of included studies about prevention.

Autor (date) and country	Study design (follow up)	Sample size and gender (cases and controls)	Age of subjects (in years) or (mean ±standard deviation in years)	Procedures	Periodontal parameters (calibration)	Statistics	Main results	Quality assessment or risk of bias
Stabholz et al. 1991 [34]—Israel	Longitudinal study (3 weeks)	Case: 30 institutionalized DS patients; Control: Absence	Case: 8–13; Control: absence	Group I (n = 10)- teeth coated with sustained-release delivery system of chlorhexidine (CHX); Group II (n = 10)- teeth coated with placebo solution; Group III (n = 10)- control (no intervention); Application twice a week for 21 days	PI [35]; GI [36] Number of papillae that bled upon gentle insertion of wooden toothpick interproximally (% bleeding); Index teeth (permanents); Calibration: Absence	Kruskal-Wallis, ANOVA, Mann-Whitney	Group I- reduction of PI, GI and % bleeding; no statistical difference compared to Group II; Group I and II- reduction in clinical parameters with statistical difference compared to Group III	Medium quality
Shapira & Stabholz, 1996 [37]—Israel	Longitudinal study (30 months)	Case: 20 DS institutionalized children (9 male/ 11 female); Control: absence	Case: 8–13; Control: absence	Elimination of dental biofilm (oral hygiene instruction, supra- and subgingival scaling), application of topical fluorides and fissure sealants. Periodontal maintenance treatment every four months (7 times)	PI [35]; GI [36]; Number of papillae that bled upon gentle insertion of wooden toothpick interproximally (% bleeding); Index teeth (permanents); Calibration: Absence	Wilcoxon	After 30 month-period, reduction of PI, GI and % bleeding, with statistical difference for PI and GI (p < 0.01)	Medium quality
Shyama et al, 2003 [7]–Kuwait	Longitudinal study (3 months)	Case: 112 DS patients (67 females/ 45 males); Control: absence	Case: 11–22 (±14.8); Control: absence	School-based supervised tooth brushing program with dental health posters, classroom activities, videotapes, dental health slogans and supervised tooth brushing; No professional prophylaxis.	PI [35];GI [36];4 sites per tooth/full mouth;Calibration: The inter-examiner for plaque (r = .96) and gingivitis scores (r = .94)	Paired t-test, t test, ANOVA, Pearson’s correlation.	Significant reduction in PI and GI in all subjects (p<0.001).	Low quality
Teitelbaum et al, 2009 [38]—Brazil	Cross-over clinical trial	Case: 40 institutionalized DS patients; Control: absence	Case: 7–13;Control: Absence	Group I (fluoridated dentifrice); Group II (fluoridated dentifrice + CHX); Group III (fluoridated dentifrice + CHX + plaque-disclosing agent); Group IV (fluoridated dentifrice + plaque-disclosing agent); Experimental period 10 days, 15-day washout; Instructions on oral hygiene an orientation for parents and patients.	GI [39]; Index of plaque [40]; Index teeth; Calibration: kappa intra-examiner: (GI) = 0.93; (PI) = 0.88	Friedman test with Dunn post hoc test, Cochran test, Wilcoxon and McNemar	The comparison (intra-group) among the indices of initial and final PI and GI showed significant differences in all the groups (P < 0.001); PI reduction in all groups: Group I (15%) Group II (11%), Group III (64%) and Group IV (65%). Significant differences (p < 0.001) between Group III and Group IV for Group I and Group II; GI significant differences were observed among the groups (p < 0.001) with reduction the Group I (8%), Group II (21%), Group III (37%) and Group IV (18%).	Unclear risk of bias
Freedman et al, 2011 [41]—Ireland	Cross-over randomized trial (24 months)	Case: 27 DS patients, (15 females/ 12 males); Control: absence	Case: 9.2–43.1 (±25.4); Control: absence	Phase 1: 1% CHX varnish every 3-months+ 3 monthly prophylaxis+ 1% CHX gel at-home daily Phase 2: 40% CHX varnish every 6-months+ 6 monthly prophylaxis+ 1% CHX gel at-home daily Control phase: 6 monthly prophylaxis+ 1% CHX gel at-home daily.Experimental period 12 months, 3-month washout. Patients, parents and caregivers were instructed on oral hygiene and topical application of CHX. Evaluation with questionnaire.	Modified gingival index [42]; Gingival-bleeding index [43]; Calculus index [44]; Plaque index [45]; Pocket probing depths Index sites [46]; Calibration: absence.	Student’s t-test with matched pairs	There were significantly lower mean pocket probing depths and modified gingival indices for the control phase compared to phase one. There were significantly lower mean gingival bleeding indices for phase two compared to the control phase.	Unclear risk of bias

DS, Down syndrome; BOP, bleeding on probing; CHX, chlorhexidine; CD, cerebral palsy; PD, probing depth; CAL, clinical attachment level; BL, bone loss; PI, plaque index; GI, gingival index; SRP, scaling and root planing

**Table 2:** Characteristics of included studies about treatment.

Author (date) and country	Study design (follow up)	Sample size and gender (cases and controls)	Age of Subjects (in years) or (mean ±standard deviation in years)	Procedures	Periodontal parameters (calibration)	Statistics	Main results	Quality Assessment or risk of bias
Cichon et al., 1998 [8]—Germany	Controlled clinical trial (12 weeks)	Case: 10 DS patients (4 females/ 6 males); Control: 11 patients with cerebral palsy (CD) (4 females/ 7 males)	Case: 20–31; Control: 23–53	Professional tooth cleaning and oral hygiene—instructions (only in Baseline)	PI [35]; GI [36]; PD; CAL; 4 sites per tooth/ full mouth; Calibration: absence	Wilcoxon signed-rank test	Clinical examinations 1, 4 and 12 weeks; Mean PI, GI scores; PD and CAL remained unchanged; Mean percentage (%) of sites with bleeding on probing (BOP) and with PD<3mm, 4-6mm, and >7mm during course of trial with no improvement	High risk of bias
Sakellari et al., 2001 [47]—Greece	Longitudinal study (6 months)	Case: 5 DS patients (2 females/ 3 males); Control: absence	Case: 26–37; Control: absence	Professional tooth cleaning (twice SRP and every 6 weeks professional prophylaxis as 0.2% CHX mouthrinse solution to be used once daily) and oral hygiene instructions for patients and caregivers	PD; Probing attachment level (PAL); BOP; Hygiene index (presence or absence of plaque)—6 sites per tooth/full mouth; Calibration: present, but not related	Paired t-test	Reduction of PD, BOP and plaque after 3 months, with the exception of PAL; No difference in clinical parameters between the 3 and 6 months; Significant plaque reduction at 6 months, although 60% of sites were still positive for plaque	Medium quality
Zaldivar-Chiapa et al., 2005 [48]—Mexico	Split-mouth study—(1 year)	Case: 14 DS patients, (5 females/ 9 males) Control: absence	Case: 17–30—Control: absence	Surgical (open flap debridement) and non-surgical (SRP) periodontal therapies; Polishing weekly for 8 weeks, after that maintenance every 2 weeks for 4 months and once a month until completing 1 year	PI [35]; GI [36]; PD; CAL; 6 sites per tooth/full mouth; Calibration: absence	Paired t test	Significant reduction in PI, GI and PD with both types of therapies (p<0.001); PDs of 1- 3mm were statistically significantly greater with non-surgical methods; Surgical treatment showed greater reduction in PDs for pockets >4mm	High risk of bias
Cheng et al., 2008 [49]—China	Longitudinal case series (12 months)	21 DS patients, (7 females/ 14 males) Control: absence	Case: 25.3±5.5; Control: absence	Oral hygiene instruction for DS patients and parents/guardians; Non-surgical mechanical periodontal therapy (SRP) followed by monthly recalls and the adjunctive use of CHX gel for brushing and CHX mouthwash twice daily	Presence of plaque; BOP; PD; CAL; 6 sites per tooth/full mouth; Calibration: absence		Deep pockets (≥7 mm): Plaque decreased from 99.1% to 38.4%; BOP 93.9% to 40.2%; mean PD decreased from 7.5 to 2.6 mm, with a mean gain in CAL of 2.9 mm; Moderately deep pockets (4 to 6 mm): Plaque decreased from 93.7% to 35.2%; BOP 93.1% to 37.9%; mean PD decreased from 4.5 to 2.2 mm, with a 1.1-mm gain in attachment level; Shallow sites (≤3 mm): Plaque decreased from 80.0% to 18.4%; BOP 76.2% to 26.7%; mean PD decreased from 2.4 to 1.6mm, with a 0.3-mm gain in attachment level	Medium quality

DS, Down syndrome; BOP, bleeding on probing; CHX, chlorhexidine; CD, cerebral palsy; PD, probing depth; CAL, clinical attachment level; BL, bone loss; PI, plaque index; GI, gingival index; SRP, scaling and root planing

## Results

**Fig 2:**
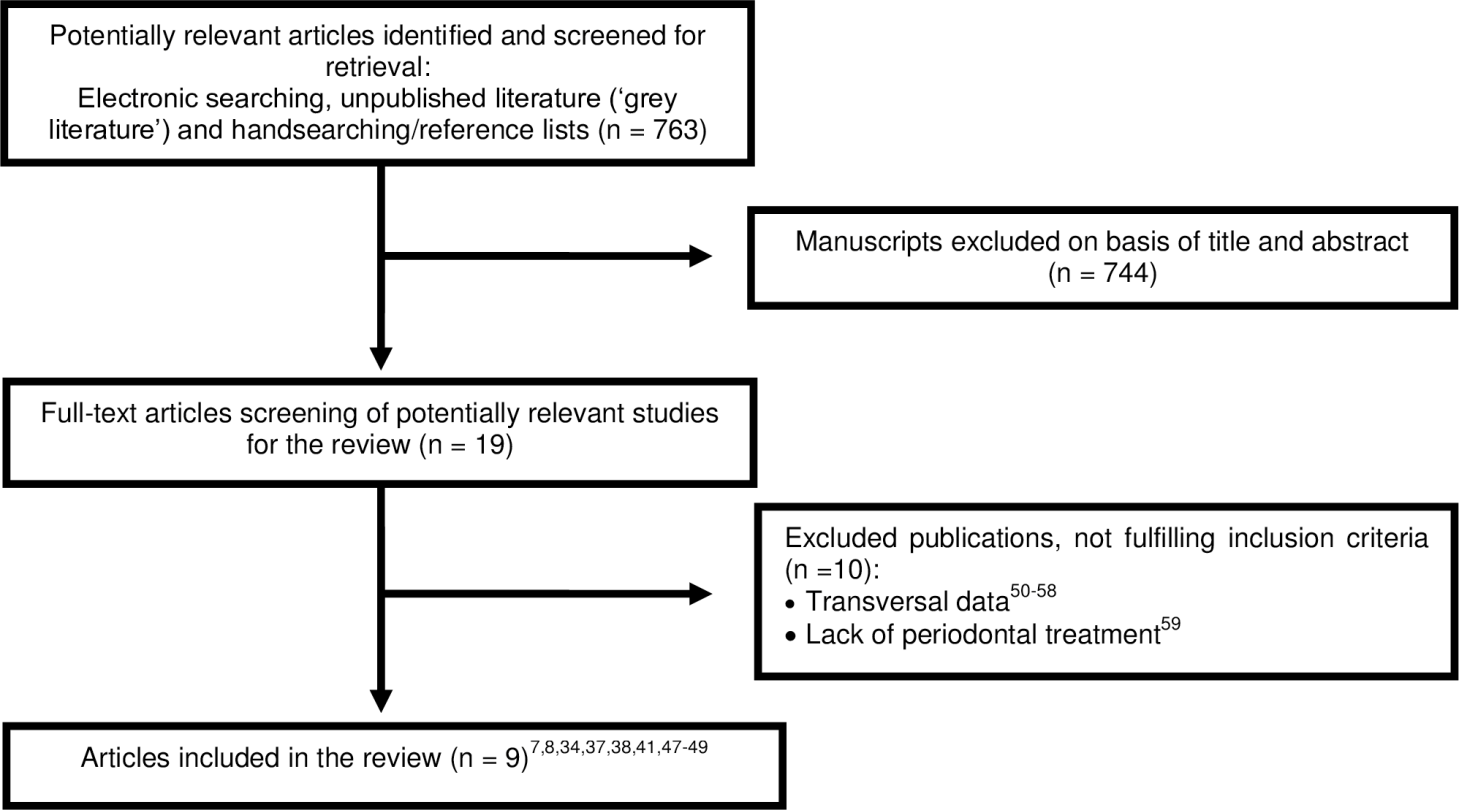
Flowchart of manuscripts screened trough the review process.

Electronic and manual search resulted in 763 papers, and of them 744 were excluded after title/abstract assessment. The full text of 19 potentially eligible publications was screened and 9 studies meet inclusion criteria (Fig 2). Among these nine papers, four was longitudinal studies [7,34,37,47], one prospective case series [49] and four clinical trials, including two cross-over studies [38,41], one controlled clinical trial [8] and one split-mouth [48]. Characteristics of included studies are presented in Tables 1 and 2. The Kappa values for inter-reviewer agreement for study inclusion were 0.87 (0.75, 0.99) for titles and abstracts and 1 for full-text articles, indicating strong agreement. Among excluded studies, nine presented periodontal parameters just in one moment (transversal evaluations) [50–58] and one study performed only data evaluation without presenting a periodontal treatment or preventive programs [59].

### Preventive programs and periodontal treatments

Different forms of preventive programs and periodontal treatments of each study are described in Table 3. Three studies evaluated [8,37,47,49] DS patients’ outcomes after sessions of scaling and root planing. One study [48] compared surgical and non-surgical periodontal therapies. Another study evaluated the effect of a supervised toothbrushing program [7]. Six studies [8,34,38,41,47–49] considered different forms and uses of chlorhexidine (CHX). Three studies [34,38,41] aimed to evaluate the effect of CHX on periodontal status of DS patients, while one of them [38] added a plaque disclosing agent in two groups. Another three studies [47–49] employed CHX as adjuvant in periodontal treatment. CHX mounthrinses (0.12% and 0.2%) and 1% CHX gel daily application presented positive outcomes [49]. Participation of parents, caregivers and institutional attenders and DS patients motivation were encouraged in all studies with the exception of one study [34]. The frequency of professional attendance was highly variable and two studies [7,38] cited no professional oral intervention, but just an educational program.

**Table 3:** Characteristics of preventive programs and periodontal therapies.

Study/ Characteristics	Stabholz et al., 1991 [34]	Shapira & Stabholz, 1996 [37]	Cichon et al., 1998 [8]	Sakellari et al., 2001 [47]	Shyama et al., 2003 [7]	Zaldivar-Chiapa et al., 2005 [48]	Cheng et al., 2008 [49]	Teitelbaum et al., 2009 [38]	Freedman et al., 2011 [41]
Intervention	Use of CHX/placebo/ control group	Professional care (supra- and subgingival scaling)	Professional care (supra- and subgingival scaling)	Professional care (supra- and subgingival scaling)	Supervised toothbrushing program	Surgical/ Non-surgical periodontal therapies	Professional care (scaling/root debridement)	Use of CHX dentifrice/ plaque disclosing agent	Use of CHX gel and CHX varnish
Parents/ Caregivers/ Institutional Attenders Participation	-	+	+	+	+	+	+	+	+
DS Patients Motivation	-	+	+	+	+	+	+	+	+
Frequency of Professional Attendance	2x/week	four months	baseline	Each 6 weeks	2x/week	1x/week (2- month); 2x/month (4-month); 1x/month (until 1-year)	1x/month	-	3 and 6 months
Use of Adjunctive Chemical Agent	Sustained release delivery system CHX	-	-	0.2% CHX mouthrinse (only in baseline)	-	0.12% CHX mouthrinse (first 8 weeks)	0.2% CHX mouthrinse/ 1%CHX gel (12 month)	CHX dentifrice/ plaque disclosing agent (3x/day)	1% CHX gel/ 1% CHX varnish/ 40% CHX varnish
Periodontal Outcome Evaluation	+ (CHX and placebo)	+ (statistical difference)	-	+ (until 3-month), no difference in 6-month evaluation	+ (statistical difference), better outcomes in youngest subjects	+(statistical difference) for both therapies	+	+(plaque disclosing agent/CHX with statistical difference)	+(1% CHX gel)

DS, Down syndrome; +, presence/positive; -, absence/negative

### Clinical parameters and outcomes

A total of 279 DS patients were evaluated including institutionalized and home patients. Individual studies presented different methods of analysis, thus data was considered too heterogeneous to be included into pooled estimates. Considering primary outcomes, all studies included assessment of different plaque and gingival indices. Five studies [8,41,47–49] also included periodontal parameters related to probing depth (PD) and four studies considered clinical attachment level (CAL) [8,47–49] in evaluation of implemented therapies. Studies could demonstrate reduction in plaque and gingival indices, with the exception of Cichon et al. (1998) [8]. Parameters remained unchanged after professional tooth cleaning and oral hygiene instructions, though procedures were executed only at baseline without any periodontal assistance during 12-week of the experiment [8]. The frequency of assistance for DS patients is important for a healthy oral condition, professional intervention and periodontal maintenance significantly reduced plaque and gingival indices, irrespective of the treatment performed [37,47–49]. The use of CHX in different forms and concentrations also reduced these parameters [34,38,41] and plaque disclosing agents presented superior outcomes compared to CHX in plaque reduction [38]. The basic regime of CHX gel (1%) applied topically on a daily basis may offer a greater improvement on markers of periodontal disease when compared to this regime supplemented with the application of CHX varnish [41]. The study of Shyama et al. (2003) [7]included a preventive program (supervised toothbrushing) without professional oral intervention conducted twice a week. It was observed significant reduction in PI and GI with superior outcomes for the youngest age group. Among studies that assessed periodontal parameters, PD [8,41,47–49] and CAL [8,47–49], four [41,47–49] observed reduction in PD and two [48–49] noticed CAL gain. In the study of Zaldivar-Chiapa et al. 2005 [48], PD of 1- 3mm were statistically significantly improved with non-surgical in comparison with surgical methods. However, surgical treatment showed greater reduction in PDs than non-surgical therapy for pockets > 4mm. None of the included studies evaluated radiographic bone loss.

### Quality assessment and risk of bias in the included trials

Among prospective observational studies, including one case series, 4 were of medium quality [34,37,47,49], and one of low quality [7]. Among clinical trials considered in this review, two presented an unclear risk of bias [38,41] and another two, high risk of bias [8,48].

## Discussion

### Summary of main findings

The results demonstrated the importance to introduce youngest DS patients in preventive programs, as well as participation of parents, caregivers or institutional attendants in supervising/performing oral hygiene. In studies with higher frequency of attendance, all age groups presented superior preventive and therapeutic results, irrespective of the therapeutic approach used (surgical/nonsurgical/periodontal care program). The important factors for reducing periodontal parameters were the frequency of the appointments and association with CHX/plaque disclosing agents as adjuvant treatment.

### Quality of the evidence and potential biases in the review process

In the present review, most of the observational studies (80%) presented medium methodological quality. Fifty percent of clinical trials presented unclear risk of bias and 50% high risk of bias. Studies were included if periodontal parameters were evaluated initially and at a follow up (longitudinal study/ interventional study). Lack of longitudinal evaluation and description of periodontal treatment/dental care program were reasons for exclusion of studies. Initial establishment of the study protocol, search with no language restrictions, independent and duplicate screening of studies in different databases limited the effect of potentially bias. Small number of studies and quality of evidence are potentially bias of this review. Factors related to studies designs may also impair additional detailed data analysis. A narrative synthesis of the findings from the included studies was performed. Substantial heterogeneity regarding participants, methodology, periodontal prevention approaches, treatment and outcomes were observed. Then, a quantitative synthesis (meta-analysis) could not be executed. It would be relevant if interventions performed in DS patients were compared to patients without syndrome and/or with special needs (control group) [8]. The wide variety of periodontal indices adopted often with partial evaluation (index teeth) [34,37,38,41] may also have influenced the outcomes. However, this systematic review permitted to point out some important and relevant aspects in the management of DS patients to prevent and/or treat periodontal disease.

### Agreements and disagreements with previous studies/reviews

Systematic reviews about DS patients are scarce as well as clinical trials and even observational studies. Studies about periodontal diseases in these patients demonstrated rapid progression even in the younger age groups [8,9]. In the study of Shyama et al. 2003 [7], youngest age group presented more reduction of plaque and gingivitis scores compared to older age groups. Clearly, younger patients seem to present more positive attitude regarding supervised toothbrushing program compared to older patients [7]. Additionally, older patients demonstrated inferior practical skills [7]. In accordance with our results, these two factors emphasize the importance of early preventive approaches in DS patients.

In agreement with our review, participation of family members, caregivers and institutional attendants are essential components in the periodontal treatment or prevention programs [60]. According to review of Frydman & Nowzari (2012) [60], cognitive deficiencies and reduced manual capacity to perform satisfactory dental hygiene should encourage more participation of family members/caregivers with this responsibility. Both DS patients and their caregivers should receive oral hygiene instruction [7,8,37,38,41,47–49,60]. Systematic review of Anders & Davis (2010) [61] reported that impaired physical coordination and cognitive skills limit the ability of DS patients to independently perform sequential tasks such as daily tooth brushing. Thus, oral hygiene procedures are dependent of knowledge, attitude and supervision of a responsible person. However, many caregivers receive minimal training to assist DS patients in oral hygiene care. Furthermore, absence of proper supervision and negative attitudes toward dental health by the caregiver has been cited as obstacles to good oral health [62]. Two studies [8,48] of the present systematic review considered active participation of parents and caregivers as inclusion criteria. These efforts should also be extended to the school or institution environment. Teachers and institutional attendants should be prepared to early introduce disabled school-age children with effective methods to improve dental health. With this goal, establishment of education programs for teachers, use of alternative materials and methods and again inclusion of the family and caregivers in dental health programs are essential [63]. Trained and qualified special education teachers can incorporate oral hygiene maintenance for children with disabilities into the daily classroom routine [64]. Patients with disabilities can learn and perform toothbrushing procedures by themselves once are encouraged and motivated [7]. According to Shyama et al. (2003) [7], during study period, most of DS patients improved their motor capability and dexterity in brushing their tooth and developed self-care skills [7]. Use of alternative materials and methods (psychological support and social reinforcements) by dental hygienists and teachers seemed to demonstrate a positive and strong effect on these individuals, improving their attitude about dental hygiene procedures [7].

Professional local treatment and maintenance program associated with a rigorous home oral hygiene regimen are the key elements to assure an effective control of the disease in patients with special needs [48]. In accordance to review of Frydman & Nowzari (2012) [60], scaling and root planing as a primary therapy should be initiated early for patients with disabilities and with higher frequency. Cichon et al. (1998) [8] and Hanookai et al. (2000) [65] did not observe any improvement in clinical and microbiological parameters after a single session of scaling and root planing and oral hygiene instructions. However, data from Sakellari et al. (2001) [47] suggested that a frequent recall program could overcome these problems [47]. Professional dental approaches are effective for reduce probing depth, plaque and bleeding indexes, but are impractical to be performed daily [34,37]. Therefore, depending on periodontal condition, physical coordination, cognitive skills and participation of parents/caregivers, ideal frequency of assistance must be defined.

Although several efforts are made to improve oral hygiene pattern in patients with disabilities, frequently mechanical actions solely are insufficient [8]. This emphasizes the importance of an association between mechanical and chemical control of the dental biofilm in DS patients [38,49]. Among different chemical agents, CHX demonstrated reduction in plaque bacteria by up to 62% [34], control of dental biofilm and reduction of gingival bleeding [38]. In this review different forms and concentrations of CHX were used, but literature presents lack of information related to this specific agent for DS patients. Stabholz et al. (1991) [34] related similar periodontal outcomes using a sustained-release delivery system of CHX and placebo, as well as observed in a systematic review [66]. Authors reported that locally delivered CHX presented a modest effect on non-surgical periodontal therapy [66] and CHX mouthwash was considered the gold standard for chemical plaque control [67]. Another study [68] demonstrated that patients with disabilities could benefit from CHX rinsing in terms of plaque reduction, but the plaque index fluctuated at every examination and finally was similar to baseline. Thus, the role of single use of CHX mouthwash in the mentally handicapped subjects to reduce plaque adequately is questionable. One study [49] demonstrated that association of CHX as a twice-daily mouthrinse and in a gel for toothbrushing, in place of a regular dentifrice, seems to have a positive impact on plaque and gingival inflammation levels in those with poor oral hygiene. In our systematic review, both CHX mounthrinses (0.12% and 0.2%) and 1% CHX gel daily application were effective for DS individuals [41,48,49]. An association of 1% CHX gel daily application and CHX varnish (1% and 40%) varnish demonstrated no expressive advantages. The use of 40% CHX varnish suggested a greater eating difficulty due to altered taste sensation^41^. Nevertheless, considering positive outcomes of CHX, this agent may offer an effective preventive and therapeutic regimen for patients with disabilities [69].

Another chemical substance with fundamental role in motivation is plaque disclosing agents [38]. The study of Teitelbaum et al. (2009) [38] demonstrated that use of a plaque disclosure agent presented greater reduction of the dental biofilm compared to fluoridated dentifrice plus chlorhexidine. This substance clearly provides identification of dental biofilm by patients, parents and/or caregivers, particularly in areas where removal by oral hygiene procedures are more difficult. Therefore, patients were possibly motivated to brush more thoroughly [38].

The systematic review of Anders & Davis (2010) [61] described relevant information about oral health of patients with intellectual disabilities and stressed the need for further research. According to authors three main areas should be considered: development of strategies to (1) increase patient acceptance of routine periodontal and restorative dental care, (2) ensure that dentists and hygienists are prepared to provide this care, and (3) to minimize the need for this care with effective preventive procedures. Dental health provides a huge impact on social acceptance and quality of life.

### Implications for research

Considering the high prevalence and severity of periodontal disease in DS individuals, more researches about comprehensive periodontal care are necessary. Recurrent failure of mechanical plaque control procedures by DS patients predicts the continuation of further investigation efforts to evaluate the efficacy of adjunctive chemical agents. Furthermore, development of effective prevention programs for DS patients, mainly younger individuals, is the greatest opportunity to improve oral health. Research in this area should focus on strategies to encourage self-care and to stimulate daily hygiene procedures performed and supervised by caregivers.

### Implications for clinical practice

DS patients represent a significant number of the population with ascending life expectancy. Therefore, they will be frequent patients for periodontal assistance on private practice and institutions. Additionally, this condition is highly associated with severe and generalized periodontal disease, with rapid progression [8–16]. Then, information regarding preventive and therapeutic approaches is essential for their oral health. This systematic review can contribute as an important updated guide for practitioners. The ideal approach to control and maintain periodontal health of DS patients is the involvement of dental professional, family/caregiver, school or institution and DS individual properly [7,8,37,38,41,47–49,60]. Professionals must search for information and guidelines related to special care of DS patients. Frequency of attendance, instruction in oral hygiene and continuous motivation are more important than therapeutic procedure selected. Use of plaque disclosing agents and CHX may contribute to plaque control, since recurrent failures in mechanical control are observed [8,38,49]. Moreover, data indicated significant effect of participation of parents, caregivers or institutional attendants in supervising/performing oral hygiene of these patients.

## Conclusions

This systematic review demonstrated the importance to early introduce DS patients into preventive programs and periodontal therapy. Thus, the participation of parents, caregivers or institutional attendants in supervising/performing oral hygiene is essential for prevention and control of the periodontal disease. In addition, frequency of attendance and association with chemical adjuvants (independently of the periodontal treatment adopted) seems to improve periodontal outcomes in preventive and periodontal treatment of DS patients. More clinical trials about preventive and periodontal treatment in DS patients are needed, including antimicrobial agents and other adjuvant treatments.

## Supporting Information

S1 PRISMA Checklist PRISMA Checklist. (DOC)

S1 Table List of titles selected for full-text analysis and the reasons for inclusion. (DOCX)

S2 Table List of titles selected for full-text analysis and the reasons for exclusion. (DOCX)
