# Supplementary material for: Prevention and Periodontal Treatment in Down Syndrome Patients: A Systematic Review
Source: PLoS One. 2016 Jun 29;11(6):e0158339. doi: 10.1371/journal.pone.0158339 (PMC4927059; doi:10.1371/journal.pone.0158339)
Supplement: S1 Table — (DOCX) [file pone.0158339.s002.docx]

**List of titles selected for full-text analysis and the reasons for inclusion.**

| **Reference** | **Classification** |
| --- | --- |
| Stabholz A, Shapira J, Shur D, Friedman M, Guberman R, Sela MN. Local application of sustained-release delivery system of chlorhexidine in Down’s syndrome population. *Clin Prev Dent* 1991;13:9-14. | Presented all inclusion criteria determined (longitudinal study, prevention approaches and at least 2 clinical parameters in different times). |
| Shapira J, Stabholz, A. A comprehensive 30-month preventive dental health program in a pre-adolescent population with Down´s Syndrome: a longitudinal study. *Spec Care Dent* 1996;16(1);33-7. | Presented all inclusion criteria determined (longitudinal study, prevention approaches and at least 2 clinical parameters in different times). |
| Shyama M, Al-Mutawa, SA, Honkala S, Honkala E. Supervised toothbrushing and oral health education program in Kuwait for children and young adults with Down syndrome. *Spec Care Dent* 2003;23(3):94-9. | Presented all inclusion criteria determined (longitudinal study, prevention approaches and at least 2 clinical parameters in different times). |
| Teitelbaum AP, Pochapski MT, Jansen JL, Sabbagh-Haddad A, Santos FA, Czlusniak GD. Evaluation of the mechanical and chemical control of dental biofilm in patients with Down syndrome. *Community Dent Oral Epidemiol* 2009; 37: 463–467. | Presented all inclusion criteria determined (Cross-over clinical trial, prevention approaches and at least 2 clinical parameters in different times). |
| Freedman L, Nunn J, Thomas W, Claffey N and Kelly A. Preventive strategies to improve periodontal health in people with Down syndrome. *Journal of Disability and Oral Health* 2011;12(2):59-67. | Presented all inclusion criteria determined (Cross-over clinical randomized trial, prevention approaches and at least 2 clinical parameters in different times). |
| Cichon P, Crawford L, Grimm WD. Early-Onset Periodontitis Associated With Down's Syndrome-A Clinical Interventional Study. *Ann Periodontol* 1998;3:370-80. | Presented all inclusion criteria determined (Controlled clinical trial, periodontal treatment and at least 2 clinical parameters in different times). |
| Sakellari D, Belibasakis G, Chadjipadelis T, Arapostathis K, Konstantinidis A. Supraginigval and subgingival microbiota of adult patients with Down’s syndrome. Changes after periodontal treatment. *Oral Microbiol Immunol* 2001;16:376–382. | Presented all inclusion criteria determined (longitudinal study, periodontal treatment and at least 2 clinical parameters in different times). |
| Zaldivar-Chiapa RM, Arce-Mendoza AY, De La Rosa-Ramírez M, Caffesse RG, Solis-Soto JM. Evaluation of Surgical and Non-Surgical Periodontal Therapies, and Immunological Status, of Young Down’s Syndrome Patients. *J Periodontol* 2005;76:1061-1065. | Presented all inclusion criteria determined (split-mouth study, periodontal treatment and at least 2 clinical parameters in different times). |
| Cheng RH, Leung WK, Corbet EF. Non-surgical periodontal therapy with adjunctive chlorhexidine use in adults with down syndrome: a prospective case series. J Periodonto. 2008 Feb;79(2):379-85. | Presented all inclusion criteria determined (longitudinal study, prevention approaches and at least 2 clinical parameters in different times). |
