# Supplementary material for: Prevention and Periodontal Treatment in Down Syndrome Patients: A Systematic Review
Source: PLoS One. 2016 Jun 29;11(6):e0158339. doi: 10.1371/journal.pone.0158339 (PMC4927059; doi:10.1371/journal.pone.0158339)
Supplement: S2 Table — (DOCX) [file pone.0158339.s003.docx]

***List of titles selected for full-text analysis and the reasons for exclusion.**

| **Reference** | **Classification** |
| --- | --- |
| Amano A, Kishima T, Kimura S, Takiguchi M, Ooshima T, Hamada S, Morisaki I. Periodontopathic bacteria in children with Down syndrome. J Periodontol 2000;71(2):249-55. | Excluded: No description of periodontal treatment or preventive approaches; transversal study including microbiology analysis. |
| Reuland-Bosma W, van der Reijden WA, van Winkelhoff AJ.Absence of a specific subgingival microflora in adults with Down's syndrome. J Clin Periodontol 2001;28(11):1004-9. | Excluded: No description of periodontal treatment or preventive approaches; transversal study including microbiology analysis. |
| López-Pérez R, Borges-Yáñez SA, Jiménez-García G, Maupomé G. Oral hygiene, gingivitis, and periodontitis in persons with Down syndrome. Spec Care Dentist 2002;22(6):214-20. | Excluded: No description of periodontal treatment or preventive approaches; transversal study. |
| Pels E, Mielnik-Błaszczak M. Oral hygiene in children with Down's syndrome. Ann Univ Mariae Curie Sklodowska Med 2002;57(2):442-7. | Excluded: No description of periodontal treatment or preventive approaches; transversal study. |
| Bagić I, Verzak Z, Cuković-Cavka S, Brkić H, Susić M. Periodontal conditions in individuals with Down's syndrome. Coll Antropol 2003;27(2):75-82. | Excluded: No description of periodontal treatment or preventive approaches; transversal study. |
| Allison PJ, Lawrence HP. A paired comparison of dental care in Canadians with Down syndrome and their siblings without Down syndrome. Community Dent Oral Epidemiol 2004;32(2):99-106. | Excluded: No description of periodontal treatment or preventive approaches; transversal study with focus in dental caries. |
| Sakellari D1, Arapostathis KN, Konstantinidis A. Periodontal conditions and subgingival microflora in Down syndrome patients. A case-control study. J Clin Periodontol 2005;32(6):684-90. | Excluded: No description of periodontal treatment or preventive approaches; case-control study including microbiology analysis. |
| Yoshihara T, Morinushi T, Kinjyo S, Yamasaki Y. Effect of periodic preventive care on the progression of periodontal disease in young adults with Down's syndrome. J Clin Periodontol 2005;32(6):556-60. | Excluded: Data were not clearly specified (baseline and post treatment) in different periods. |
| Zigmond M, Stabholz A, Shapira J, Bachrach G, Chaushu G, Becker A, Yefenof E, Merrick J, Chaushu S. The outcome of a preventive dental care programme on the prevalence of localized aggressive periodontitis in Down's syndrome individuals. J Intellect Disabil Re. 2006;50(7):492-500. | Excluded: No description of periodontal treatment or preventive approaches; transversal study. |
| Agholme, M. B., Dahllöf, G. and Modéer, T. Changes of periodontal status in patients with Down syndrome during a 7-year period. European Journal of Oral Sciences 1999;107:82–88. | Excluded: No description of periodontal treatment or preventive approaches. |
